# Cost-Effectiveness and Cost Thresholds of Generic and Brand Drugs in a National Chronic Hepatitis B Treatment Program in China

**DOI:** 10.1371/journal.pone.0139876

**Published:** 2015-11-04

**Authors:** Mehlika Toy, David W. Hutton, Samuel K. So

**Affiliations:** 1 Asian Liver Center, Department of Surgery, Stanford University School of Medicine, Stanford, CA, 94305, United States of America; 2 Department of Health Management and Policy, University of Michigan, Ann Arbor, MI, 48109, United States of America; Yonsei University College of Medicine, REPUBLIC OF KOREA

## Abstract

Chronic liver disease and liver cancer associated with chronic hepatitis B (CHB) are leading causes of death among adults in China. Although newborn hepatitis B immunization has successfully reduced the prevalence of CHB in children, about 100 million Chinese adults remain chronically infected. If left unmanaged, 15–25% will die from liver cancer or liver cirrhosis. Antiviral treatment is not necessary for all patients with CHB, but when it is indicated, good response to treatment would prevent disease progression and reduce disease mortality and morbidity, and costly complications. The aim of this study is to analyze the cost-effectiveness of generic and brand antiviral drugs for CHB treatment in China, and assessing various thresholds at which a highly potent, low resistance antiviral drug would be cost-saving and/or cost-effective to introduce in a national treatment program. We developed a Markov simulation model of disease progression using effectiveness and cost data from the medical literature. We measured life-time costs, quality adjusted life years (QALYs), incremental cost-effectiveness ratios (ICERs), and clinical outcomes. The no treatment strategy incurred the highest health care costs ($12,932-$25,293) per patient, and the worst health outcomes, compared to the antiviral treatment strategies. Monotherapy with either entecavir or tenofovir yielded the most QALYs (14.10–19.02) for both HBeAg-positive and negative patients, with or without cirrhosis. Threshold analysis showed entercavir or tenofovir treatment would be cost saving if the drug price is $32–75 (195–460 RMB) per month, highly cost-effective at $62–110 (379–670 RMB) per month and cost-effective at $63–120 (384–734 RMB) per month. This study can support policy decisions regarding the implementation of a national health program for chronic hepatitis B treatment in China at the population level.

## Introduction

China has the greatest disease burden of chronic hepatitis B (CHB) in the world, with an estimated 350,000–500,000 deaths each year from hepatitis B virus (HBV) related diseases, including hepatocellular carcinoma (HCC) and hepatic failure [1]. Approximately 80% of HCC, the most common type of liver cancer, is due to chronic HBV infection in China [2, 3]. Many Asian adults with CHB infection develop HCC at a rate of about 5% per decade, which is 100-fold higher than the rate among uninfected persons. Without monitoring or appropriate treatment, 15–25% of those chronically infected will die from liver cancer or liver cirrhosis. In comparison to HIV, which affects 600,000 Chinese, an estimated 100 million Chinese are living with chronic hepatitis B, making it the most prevalent life threatening chronic infection in China [1]. Major progress has been made in China to reduce the prevalence of chronic hepatitis B in children through a robust new born immunization program, and a recent nationwide catch up vaccination program for unprotected children [4, 5]. Although hepatitis B vaccination clearly contributed to the reduction of new cases, it does not address the healthcare needs of the chronically-infected individuals who are at risk of disease progression leading to the development of HCC and cirrhosis. There is currently no curative treatment for CHB, but good response to approved treatments could prevent disease progression and reduce deaths and costly complications.

According to both international and Chinese professional guidelines, treatment is indicated for those with chronic hepatitis B who are hepatitis B e-antigen (HBeAg)-positive and HBeAg antigen negative with active hepatitis (high HBV DNA and ALT levels) or cirrhosis. Current therapies fall into two categories: immune modulators and antiviral agents. The immune modulators such as pegylated interferon alfa are given over 6–12 months by subcutaneous injection and can induce remission of liver disease in a fraction of patients, but the remission may not be permanent. Many patients cannot tolerate interferon treatment because of the associated side effects. Antiviral agents such as nucleoside or nucleotide analogues that suppress viral replication are well tolerated and as simple as a pill a day, but likely need to be taken indefinitely. The antiviral therapies vary in terms of costs, effectiveness in suppressing viral replication and risk of drug resistance.

Despite the availability of CHB treatment, the proportion of patients actually receiving treatment is low in China [1]. The main obstacle to treatment is often the cost. In recent years, many cities have begun providing partial coverage for CHB treatment but the choice of covered treatment is limited in the rural health plans. Treatment coverage also varies in the different provinces. Currently, there is no national policy to cover CHB treatment nationwide.

This study is a comprehensive analysis of the cost-effectiveness of treatment therapies for CHB in China, and assessing various thresholds at which a highly potent low resistance drug would be cost-saving and/or cost-effective. We cover all major drugs that would be used for treatment and evaluate several different potential patient groups according to HBeAg status with or without cirrhosis. This analysis is intended to help guide discussions particularly for policy makers and health professionals about national coverage for the treatment of CHB and also provide insights into the potential cost-effectiveness of the various treatment options in China.

## Methods

### Overview

**Fig 1:**
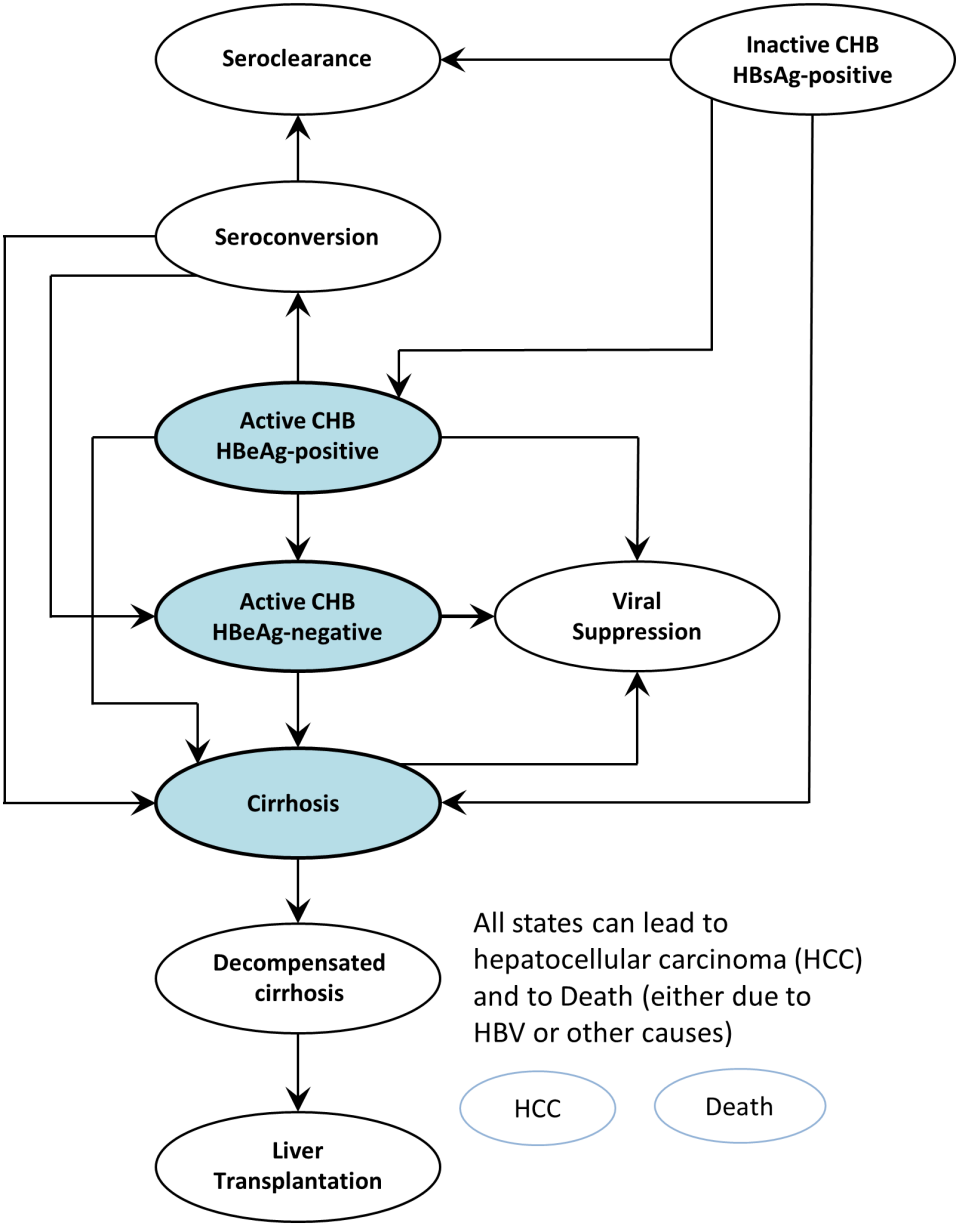
Markov model schematic with entry points active HBeAg-positive, active HBeAg-negative disease, and cirrhosis, and transition between states according to annual transition estimates.

We used a Markov model that describes disease progression to evaluate the long-term outcomes for patients under various treatment strategies. The model used is similar to our previous study on CHB in Shanghai, China [6]. Treatment-naïve, chronic HBV, HBeAg-positive or HBeAg-negative patients eligible for treatment under international treatment guidelines enter the model either in the cirrhotic or non-cirrhotic health state (Fig 1). Patients can progress to compensated cirrhosis, decompensated cirrhosis, hepatocellular carcinoma, and would be eligible to receive a liver transplantation. All patients face age-specific mortality plus increased mortality if they have cirrhosis, HCC, or a liver transplant. If patients receive treatment, they can develop drug resistance or sustained virologic response.

### Strategies

Eight different strategies were analysed in this study.

#### “No antiviral treatment.”

Chronic hepatitis B patients progress according to the natural history, following annual disease progression estimates (Table 1). The disease progression estimates were derived from recent age-specific cohort studies on inactive and active CHB in Asia [7–15]. We assumed that patients received best supportive care, except for drug treatment. Patients followed the natural history according to their HBeAg and disease status (with or without cirrhosis). Spontaneous virologic response was defined as seroconversion to anti-HBe in HBeAg-positive patients, and as persistent HBV DNA suppression and ALT normalization in HBeAg-negative patients. We assumed that a proportion of patients with decompensated cirrhosis and HCC became eligible for liver transplantation.

**Table 1:** Annual transition estimates governing natural history of chronic hepatitis B by initial state.

Transition	Age group	Estimate (%)	Range	References
**From inactive CHB, HBsAg-positive**				
To seroclearance	<30 years	0.8	(0.38–1.15)	7
	30–39 years	1.1	(0.53–1.60)	
	40–49 years	1.7	(0.82–2.47)	
	50+ years	1.8	(0.91–2.74)	
To active CHB, HBeAg-positive	<30 years	0.9	(0.4–1.3)	7,12
	30–39 years	1.4	(0.7–2.1)	
	40–49 years	2.8	(1.4–4.1)	
	50+ years	2.0	(1.0–3.0)	
To cirrhosis	<30 years	0.038	(0.019–0.057)	12
	30–39 years	0.049	(0.024–0.073)	
	40–49 years	0.068	(0.034–0.102)	
	50+ years	0.150	(0.052–0.202)	
To HCC	All ages	0.168	(0.001–0.25)	9,13,14
**From active CHB, HBeAg-positive**				
To seroconversion	All ages	7.0	(2.0–23)	8
To active CHB, HBeAg-negative	All ages	1.9	(1.0–3.8)	15
To cirrhosis	All ages	2.4	(2.1–2.6)	9
To HCC	All ages	0.8	(0.5–1.0)	9
To HBV-related death	All ages	0.6	(0.2–0.9)	9
**From active CHB, HBeAg-negative**				
To inactive CHB, HBsAg-positive	All ages	1.6	(0.0–11)	8
To cirrhosis	All ages	2.4	(1.3–3.4)	9
To HCC	All ages	0.8	(0.5–1.0)	9
To HBV-related death	All ages	0.6	(0.2–0.9)	9
**From seroconversion**				
To active CHB, HBeAg-negative	<30 years	2.9	(1.4–4.3)	14
	31–40 years	3.8	(1.9–5.7)	
	40+ years	8.6	(4.3–12.9)	
To cirrhosis	<30 years	0.2	(0.1–0.3)	
	31–40 years	1.0	(0.5–1.5)	
	40+ years	4.2	(2.1–6.3)	
To HCC	<30 years	0.1	(0.05–0.15)	
	31–40 years	0.2	(0.1–0.3)	
	40+ years	0.6	(0.3–0.9)	
To seroclearance	<30 years	0.8	(0.4–1.2)	
	31–40 years	0.7	(0.3–1.0)	
	40+ years	0.3	(0.1–0.4)	
**From seroclearance**				
To HCC	50+ years	1.0	(0.0–2.0)	11
**From cirrhosis**				
To decompensated cirrhosis	All ages	3.9	(3.2–4.6)	9
To HCC	All ages	5.0	(3.0–7.0)	
To HBV-related death	All ages	5.6	(3.1–8.0)	
**From decompensated cirrhosis**				
To liver transplantation	All ages	12.0	(6.0–18.0)	*
To HCC	All ages	7.1	(3.5–10.0)	9
To HBV-related death	All ages	15.0	(9.9–20.0)	9
**From HCC**				
To liver transplantation	All ages	4.7	(2.3–7.0)	*
To HBV-related death	All ages	54.5	(20.0–60.0)	9
**From liver transplantation**				
To HBV-related death	All ages	6.6	(2.0–12)	8

* Authors’ estimates based on consultation with experts in liver transplantation in China.

Table adapted from Toy et al. [6].

#### “Lamivudine mono-therapy” (LAM)

Patients receive 100mg orally once daily of the first licensed antiviral HBV drug. This drug is known to be associated with a high incidence of resistance [16]. Such mono-therapy is still commonly prescribed in China [17]. We assigned different rates of virologic response under long-term therapy between resistant and non- resistant patients (Table 2) [16, 18–20].

**Table 2:** Treatment transition estimates.

	Annual probability, % (range)									
Transition	Lamivudine monotherapy*		Entecavir monotherapy||		Adefovir salvage¶		Tenofovir**		Tenofovir salvage††	
	e-positive	e-negative	e-positive	e-negative	e-positive	e-negative	e-positive	e-negative	e-positive	e-negative
**From active CHB, initial treatment** †										
To sustained virologic response‡	20 (15–25)	10 (5.0–17)	22 (17–27)	11 (5.5–22)	12 (5.0–17)	10 (5.0–17)	23 (11.5–34.5)	11 (5.5–22)	19 (9.5–28.5)	11 (5.5–16.5)
To cirrhosis§	0.5 (0.2–1.0)	1.2 (0.9–2.1)	0.2 (0.1–0.5)	0.6 (0.3–1.2)	0.5 (0.2–1.0)	1.2 (0.9–2.1)	0.2 (0.1–0.5)	0.6 (0.3–1.2)	0.5 (0.25–0.75)	1.2 (0.6–1.8)
To HCC‡‡	0.2 (0.1–0.5)	0.2 (0.1–0.5)	0.2 (0.1–0.5)	0.2 (0.1–0.5)	0.2 (0.1–0.5)	0.2 (0.1–0.5)	0.2 (0.1–0.5)	0.2 (0.1–0.5)	0.2 (0.1–0.5)	0.2 (0.1–0.5)
**From active CHB, long-term treatment, drug-sensitive**										
To sustained virologic response‡	24 (19–29)	10 (5.0–17)	27 (17–27)	11 (5.5–22)	12 (5.0–17)	10 (5.0–17)	27 (17–27)	11 (5.5–22)	19 (9.5–28.5)	11 (5.5–16.5)
To cirrhosis§	0.5 (0.2–1.0)	1.2 (0.9–2.1)	0.2 (0.1–0.5)	0.6 (0.3–1.2)	0.5 (0.2–1.0)	1.2 (0.9–2.1)	0.2 (0.1–0.3)	0.6 (0.3–0.9)	0.5 (0.25–0.75)	1.2 (0.6–1.8)
To active CHB, drug resistant year 1‡	23 (18–28)	23 (18–28)	1 (0.0–2.0)	1 (0.0–2.0)	6 (1.0–12)	6 (1.0–12)	0 (0–0)	0 (0–0)	0 (0–0)	0 (0–0)
To active CHB, drug resistant year 2‡	42 (37–45)	42 (37–45)	1 (0.0–2.0)	1 (0.0–2.0)	21 (16–27)	21 (16–27)	0 (0–0)	0 (0–0)	1 (0.5–1.5)	1 (0.5–1.5)
To active CHB, drug resistant year 3‡	53 (48–58)	53 (48–58)	1 (0.0–2.0)	1 (0.0–2.0)	21 (16–27)	21 (16–27)	0.4 (0.2–0.6)	0.4 (0.2–0.6)	1 (0.5–1.5)	1 (0.5–1.5)
To active CHB, drug resistant year 4‡	70 (65–75)	70 (65–75)	1 (0.0–2.0)	1 (0.0–2.0)	21 (16–27)	21 (16–27)	0.8 (0.4–1.2)	0.8 (0.4–1.2)	1 (0.5–1.5)	1 (0.5–1.5)
To active CHB, drug resistant year 5‡	74 (69–79)	74 (69–79)	1 (0.0–2.0)	1 (0.0–2.0)	21 (16–27)	21 (16–27)	1 (0.0–2.0)	1 (0.0–2.0)	1 (0.0–2.0)	1 (0.0–2.0)
To HCC‡‡	0.2 (0.1–0.5)	0.2 (0.1–0.5)	0.2 (0.1–0.5)	0.2 (0.1–0.5)	0.2 (0.1–0.5)	0.2 (0.1–0.5)	0.2 (0.1–0.5)	0.2 (0.1–0.5)	0.2 (0.1–0.5)	0.2 (0.1–0.5)
**From active CHB, long-term treatment, drug resistant**										
To sustained virologic response‡	4.5 (3.3–7.8)	0	5 (2–7)	0.5 (0.2–1.0)	4.5 (3.3–7.8)	0	5 (2–7)	0.5 (0.2–1.0)	5 (2–7)	0.5 (0.2–1.0)
To cirrhosis	2.7 (1.6–3.8)	6.2 (2.8–9.7)	2.7 (1.6–3.8)	6.2 (2.8–9.7)	2.7 (1.6–3.8)	6.2 (2.8–9.7)	2.7 (1.6–3.8)	6.2 (2.8–9.7)	2.7 (1.6–3.8)	6.2 (2.8–9.7)
To HCC‡‡	0.4 (0.3–0.6)	0.4 (0.3–0.6)	0.4 (0.3–0.6)	0.4 (0.3–0.6)	0.4 (0.3–0.6)	0.4 (0.3–0.6)	0.4 (0.3–0.6)	0.4 (0.3–0.6)	0.4 (0.3–0.6)	0.4 (0.3–0.6)
**From cirrhosis, initial treatment** †										
To sustained virologic response‡	20 (15–25)	10 (5.0–17)	22 (17–27)	11 (5.5–22)	12 (5.0–17)	10 (5.0–17)	23 (11.5–34.5)	12 (6–18)	19 (9.5–28.5)	11 (5.5–16.5)
To HCC‡‡	0.9 (0.3–1.4)	1.5 (0.7–3.0)	0.9 (0.3–1.4)	1.5 (0.7–3.0)	0.9 (0.3–1.4)	1.5 (0.7–3.0)	0.9 (0.3–1.4)	1.5 (0.7–3.0)	0.9 (0.3–1.4)	1.5 (0.7–3.0)
**From cirrhosis, long-term treatment, drug-sensitive**										
To sustained virologic response‡	24 (19–29)	10 (5.0–17)	27 (17–27)	11 (5.5–22)	12 (5.0–17)	10 (5.0–17)	27 (17–27)	11 (5.5–22)	19 (9.5–28.5)	11 (5.5–16.5)
To cirrhosis, drug-resistant year 1	23 (18–28)	23 (18–28)	1 (0.0–2.0)	1 (0.0–10)	6 (1.0–12)	6 (1.0–12)	0 (0–0)	0 (0–0)	0 (0–0)	0 (0–0)
To cirrhosis, drug-resistant year 2	42 (37–45)	42 (37–45)	1 (0.0–2.0)	1 (0.0–2.0)	21 (16–27)	21 (16–27)	0 (0–0)	0 (0–0)	1 (0.5–1.5)	1 (0.5–1.5)
To cirrhosis, drug-resistant year 3	53 (48–58)	53 (48–58)	1 (0.0–2.0)	1 (0.0–2.0)	21 (16–27)	21 (16–27)	0.4 (0.2–0.6)	0.4 (0.2–0.6)	1 (0.5–1.5)	1 (0.5–1.5)
To cirrhosis, drug-resistant year 4	70 (65–75)	70 (65–75)	1 (0.0–2.0)	1 (0.0–2.0)	21 (16–27)	21 (16–27)	0.8 (0.4–1.2)	0.8 (0.4–1.2)	1 (0.5–1.5)	1 (0.5–1.5)
To cirrhosis, drug-resistant year 5	74 (69–79)	74 (69–79)	1 (0.0–2.0)	1 (0.0–2.0)	21 (16–27)	21 (16–27)	1 (0.0–2.0)	1 (0.0–2.0)	1 (0.0–2.0)	1 (0.0–2.0)
To decompensated cirrhosis	1.9 (0.9–3.8)	1.9 (0.9–3.8)	1.9 (0.9–3.8)	1.9 (0.9–3.8)	1.9 (0.9–3.8)	1.9 (0.9–3.8)	1.9 (0.9–3.8)	1.9 (0.9–3.8)	1.9 (0.9–3.8)	1.9 (0.9–3.8)
To HCC‡‡	1.6 (0.8–3.2)	1.6 (0.8–3.2)	1.6 (0.8–3.2)	1.6 (0.8–3.2)	1.6 (0.8–3.2)	1.6 (0.8–3.2)	1.6 (0.8–3.2)	1.6 (0.8–3.2)	1.6 (0.8–3.2)	1.6 (0.8–3.2)
To HBV-related death	2.4 (1.2–4.8)	2.4 (1.2–4.8)	2.4 (1.2–4.8)	2.4 (1.2–4.8)	2.4 (1.2–4.8)	2.4 (1.2–4.8)	2.4 (1.2–4.8)	2.4 (1.2–4.8)	2.4 (1.2–4.8)	2.4 (1.2–4.8)
**From cirrhosis, long-term treatment, drug-resistant**										
To sustained virologic response‡	4.5 (3.3–7.8)	0	5 (2–7)	0.5 (0.2–1)	4.5 (3.3–7.8)	0	5 (2–7)	0.5 (0.2–1)	5 (2–7)	0.5 (0.2–1)
To decompensated cirrhosis	7.9 (4–15)	7.9 (4–15)	7.9 (4–15)	7.9 (4–15)	7.9 (4–15)	7.9 (4–15)	7.9 (4–15)	7.9 (4–15)	7.9 (4–15)	7.9 (4–15)
To HCC‡‡	1.8 (0.9–3.8)	2.9 (1.0–5.6)	1.8 (0.9–3.8)	2.9 (1.0–5.6)	1.8 (0.9–3.8)	2.9 (1.0–5.6)	1.8 (0.9–3.8)	2.9 (1.0–5.6)	1.8 (0.9–3.8)	2.9 (1.0–5.6)
To HBV-related death	3.1 (3.1–3.8)	3.1 (3.1–3.8)	3.1 (3.1–3.8)	3.1 (3.1–3.8)	3.1 (3.1–3.8)	3.1 (3.1–3.8)	3.1 (3.1–3.8)	3.1 (3.1–3.8)	3.1 (3.1–3.8)	3.1 (3.1–3.8)

* Estimates from Kanwal 2005 [8].

^†^ Initial therapy is 12 months (48 weeks) of therapy.

^‡^ Estimates calculated by the author, based on the assumption that the natural progression rates of chronic hepatitis B are reduced by antiviral therapy. Estimates derived from natural history estimate similar to Kanwal’s assumption of no progression of disease in HBeAg seroconversion, we assume no progression of disease in case HBV DNA is undetectable by PCR. In the papers from Chang and Lai full suppression of HBV DNA was observed in 80% with a high resistance profile drug, and 90% with a low resistance profile drug. We took these percentages for our calculations [18,19].

^§^ Estimates for Lamivudine resistance from Lai et al. And Moskovitz et al. [19,20].

^||^ Estimates for Entecavir from Chang et al. 2006, Lai et al. 2006 and Colonno 2007, [18,19,25] and resistance from Colonno et al. 2006, Colonno et al. 2007 and Tenny et al. 2009. [24–26].

^¶^ Adefovir salvage resistance estimates from Lee et al., Chen et al. And Yeon et al. [21–23].

** Estimates for tenofovir from Heathcote et al. 2011 [31].

^††^ Tenofovir salvage scenario estimates from van Bommel et al., Reijnders et al., Ke et al., Gordom et al., Patterson et al., Lee et al. [32–37].

^‡‡^ Estimates based on reduction of progression rates by nucleoside analogue therapy of 50% [16].

#### “Adefovir salvage” (LAM→ADV)

Patients initially receive lamivudine, but switched to adefovir when they develop lamivudine resistance. Patients without resistance continue to receive the initial drug lamivudine [16, 21–23].

#### “Entecavir monotherapy” (ETV)

Patients in this strategy received 0.5mg entecavir once daily. The treatment related probability estimates for responding and resistant patients are shown in Table 2 [24–26]. Since entecavir and lamivudine share cross-resistance, it is not a recommended salvage therapy for lamivudine resistant patients.

#### “Pegylated Interferon” (PEG-IFN)

Patients receive 180mcg of pegylated interferon administered subcutaneously once a week for 48 weeks. If the patient does not respond or relapses in the second year after treatment, they follow the transitions in the natural history (no treatment) strategy.

#### “Pegylated Interferon, followed by entecavir” (PEG-IFN→ETV)

Patients receive 180 mcg of pegylated interferon once a week for 48 weeks. If the patient does not respond or relapses in the second year after treatment, they start entecavir at 0.5 mg/day (S1 Table) [27–30].

#### “Tenofovir monotherapy” (TDF)

Patients receive 300mg of tenofovir once daily. The annual probability of resistance in this scenario was 0% for the first and second year of treatment [31–34].

#### “Tenofovir salvage” (LAM→TDF)

In this strategy, patients who develop resistance during lamivudine monotherapy are switched to tenofovir [32, 35–37].

### Assumptions

Causes of death not related to liver disease associated with CHB were included in the model, based on age-specific mortality rates from the National Bureau of Statistics China [38]. The annual probabilities of receiving a liver transplant for decompensated cirrhosis and HCC (12% and 4.7%, respectively) in China were calculated based on data from China Liver Transplant Registry [39].

If progression rates were reported, these were transformed into annual probabilities using a standard formula (*P* = 1-*e* ^-r×t^), where *P* is the probability, *e* is the base of the natural logarithm, *r* is the event rate, and *t* is the time interval [40]. We assumed that it was possible to develop cirrhosis and hepatocellular carcinoma while on treatment, but with a 50% reduction in the rate decrease from the natural history [41].

Treatment guidelines often recommend a finite period of therapy with oral nucleoside analogs for patients with HBeAg-positive CHB who undergo HBeAg seroconversion. However, prolonged therapy is to be considered for patients with evolving HBeAg-negative CHB and active HBV DNA replication (> log^4^ IU/ml) and for patients with cirrhosis [42–46]. A more recent publication [47] suggests continuation of long-term nucleos(t)ide analogue treatment, irrespective of the occurrence of HBeAg seroconversion in HBeAg-positive patients. Following these recent findings, our model assumes continued antiviral therapy for HBeAg-positive patients even if seroconversion occurs. Also our model assumes that the resistance rate for ETV and TDF remains as low as recent studies report [33, 48]. The scenario for PEG-IFN assumes that non-responders and relapsers continue with long-term ETV treatment both in HBeAg-positive and negative patients.

### Cost and utility estimates

We used generic antiviral drug costs for the base case analysis and examined brand drug costs in the sensitivity analysis. Generic drugs are widely available in China, and whether they are prescribed often depends on the physician’s preference and the choice of the patient based on their health insurance coverage and out of pocket costs. The generic and brand drug costs were obtained from official prices approved by the Shanghai Municipal Bureau of Pricing. Prices were available for all generic antiviral drugs except for PEG-IFN and TDF which are not yet available in generic form in China. The medical management costs for CHB and other related costs were derived from the retrospective analysis of the medical records of patients with CHB by Zhiqiang et al. [49].

All costs were inflated to 2014 prices using China National Healthcare Index from the National Bureau of Statistics of China and converted to US dollars using the exchange rate as of 2014 (1 USD = 6.23 RMB). The age-specific utilities were obtained from a multinational study on hepatitis B [50] (Table 3). Following the World Health Organization guidelines for cost-effectiveness estimates [51], we regarded the incremental ratio of less than one times the gross domestic product (GDP) per capita for each QALY gained as an cost-effective intervention, and an ICER between one and three times GDP per capita per QALY as a potential cost-effective intervention, in which the GDP per capita in China in 2013 was $6,800 (41,775 RMB) [52].

**Table 3:** Annual base case cost and utility estimates (2014).

Variable	Annual base-case estimate (range)	
**Generic drug costs**	**Yuen (RMB)**	**Dollar**
Lamivudine	3,650 (2,730–4,215)	$585 (415–790)
Adefovir	3,423 (2,610–4,100)	$549 (395–751)
Entecavir (0.5mg)	7,662 (5,990–9,330)	$1,229 (972–2,003)
Pegylated Interferon	N.A	N.A
Tenofovir (300 mg)	N.A	N.A
**Branded drug costs**		
Lamivudine	4,360 (3,266–5,446)	$699 (524–873)
Adefovir	4,088 (3,060–5,108)	$656 (491–819)
Entecavir (0.5mg)	14,645 (10,984–18,306)	$2,349 (1,762–2,937)
Pegylated Interferon	69,761 (52,318–87,196)	$11,191 (8,393–13,988)
Tenofovir (300 mg)	18,202 (13,652–22,753)	$2,920 (2,190–3,650)
**Annual medical management costs**		
Annual monitoring*	126 (95–158)	$20 (15–25)
Chronic hepatitis B	1,332 (1,000–1,665)	$214 (160–267)
Cirrhosis	1,736 (1,301–2,170)	$278 (209–348)
Decompensated cirrhosis	15,974 (11,980–19,968)	$2,562 (1,922–3,203)
Hepatocellular carcinoma	44,499 (33,374–55,623)	$7,139 (5,354–8,923)
Liver transplantation	573,230 (429,923–716,538)	$91,961 (68,970–114,951)
**Health state utilities** ^**±**^	**Utility**	**Range**
Viral suppression	1	(0.95–1.00)
Seroclearance	0.99	(0.90–1.00)
Inactive CHB	0.95	(0.90–0.99)
Active CHB	0.85	(0.68–0.90)
Cirrhosis	0.69	(0.66–0.71)
Decompensated cirrhosis	0.35	(0.32–0.37)
Hepatocellular carcinoma	0.38	(0.36–0.41)
Liver transplantation	0.67	(0.64–0.69)

* Annual monitoring costs include ALT and HBV DNA tests.

^±^ See Levy et al. [50] for age-specific utilities.

### Sensitivity analysis

A Monte Carlo simulation was conducted, assuming that all variables followed a triangular distribution, with base case, low and high range values. We simulated 10,000 trials and plotted the results on willingness to pay threshold acceptability curves. We also analyzed at which threshold annual drug price at which ETV and TDF (since both these antivirals are considered highly potent with low resistance) annual drug price would be cost-effective or cost-saving. In addition we also analyzed the cost-effectiveness of branded costs for the all drugs.

## Results

### Base case

**Fig 2:**
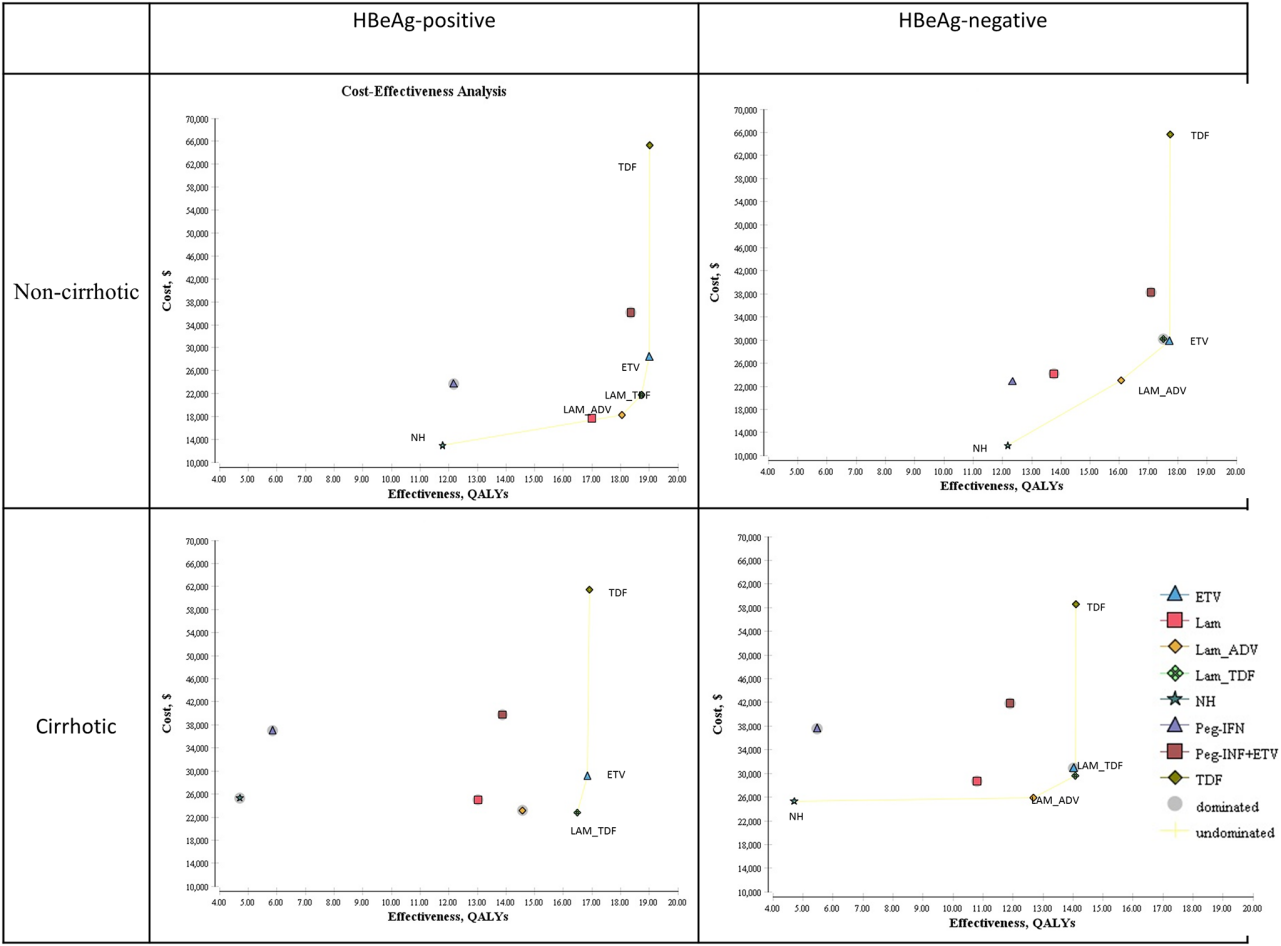
Results plotted on a cost-effectiveness plane stratified by hepatitis B e-antigen (HBeAg) with or without cirrhosis. The x-axis represents the gain in QALYs with each strategy, and the y-axis represents the total costs (year 2014 values).

**Fig 3:**
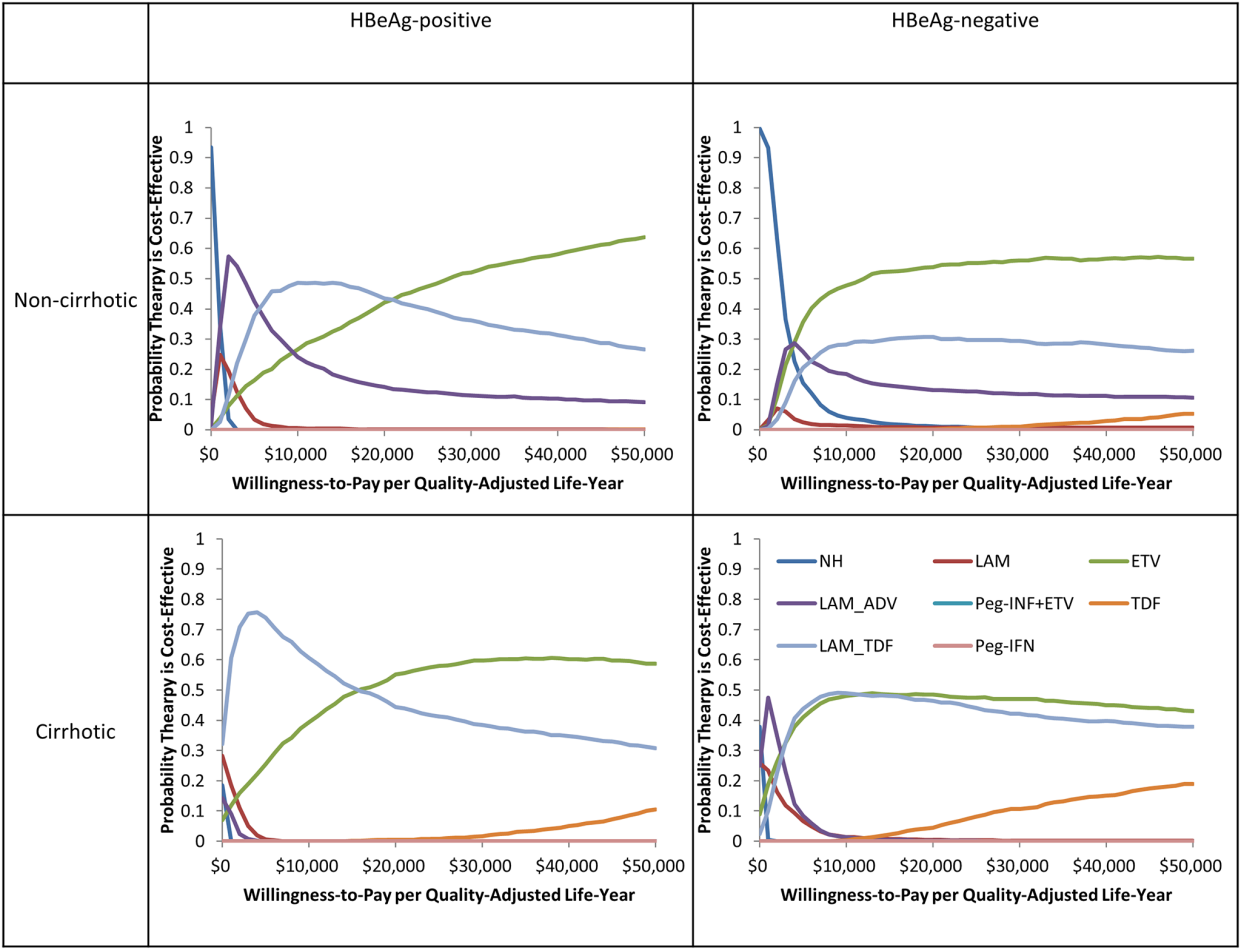
Cost-effectiveness acceptability curves showing the probabilities of net benefits achieved by each strategy for different willingness to pay thresholds stratified by hepatitis B e-antigen (HBeAg) with or without cirrhosis.

A cost-effectiveness plot of the various scenarios according to HBeAg status with or without cirrhosis is shown in Fig 2. For the base-case analysis, the lowest available prices were taken for the drugs (generic prices except for PEG-IFN and TDF, where the generic form of the drugs are not yet available in China). The CHB treatment costs, long-term health care costs, total costs, QALYs, and ICERs compared to the next best therapy for all subgroups are shown in Table 4 and for the branded price outcomes in S2 Table. The health outcomes for each sub-group in terms of morbidity (decompensated cirrhosis, hepatocellular carcinoma, and liver transplantation) and mortality are shown in Fig 3.

**Table 4:** Base Case Results.

Therapy	HBV Antiviral or Interferon Treatment Costs	Long-Term Other Healthcare Costs	Total Costs ($)	Quality Adjusted Life Years	ICER compared to next-best therapy ($/QALY)*	ICER compared to no treatment ($/QALY)**
**Non-cirrhotic HBeAg-positive**						
No Treatment	-	12,932	12,932	11.79		
(generic) Lamivudine	11,855	5,875	17,730	16.99	ext. dominated	1,042
(generic)Lamivudine with Adefovir (generic) Salvage	15,050	3,155	18,205	18.04	844	1,008
(generic) Lamivudine with (branded) Tenofovir Salvage	19,490	2,267	21,757	18.73	5,103	1,161
(generic) Entecavir	26,807	1,554	28,361	19.00	25,058	1,492
(branded) Peg-Interferon-alfa	11,191	12,174	23,365	12.35	abs. dominated	18,670
(branded) Peg-Interferon-alfa with Entecavir (generic) Salvage	33,744	2,387	36,131	18.35	abs. dominated	3,535
(branded) Tenofovir	63,720	1,518	65,238	19.02	1,900,948	3,429
**Non-cirrhotic HBeAg-negative**						
No Treatment	-	11,735	11,735	12.19		
(generic) Lamivudine	10,095	14,100	24,195	13.76	abs. dominated	1,757
(generic)Lamivudine with Adefovir (generic) Salvage	16,511	6,559	23,070	16.05	2,933	1,436
(generic) Lamivudine with (branded) Tenofovir Salvage	26,126	4,071	30,197	17.50	abs. dominated	1,725
(generic) Entecavir	26,078	3,709	29,787	17.71	4,066	1,681
(branded) Peg-Interferon-alfa	11,191	11,613	22,804	12.34	ext. dominated	71,701
(branded) Peg-Interferon-alfa with Entecavir (generic) Salvage	33,734	4,536	38,270	17.08	abs. dominated	5,426
(branded) Tenofovir	62,032	3,640	65,672	17.73	1,492,068	3,703
**Cirrhotic HBeAg-positive**						
No Treatment	-	25,293	25,293	4.70		
(generic) Lamivudine	8,530	16,490	25,020	13.02	abs. dominated	1,920
(generic)Lamivudine with Adefovir (generic) Salvage	11,015	11,752	22,767	14.64	0	1,554
(generic) Lamivudine with (branded) Tenofovir Salvage	16,214	6,580	22,794	16.48	15	1,382
(generic) Entecavir	23,498	5,606	29,104	16.84	17,497	1,727
(branded) Peg-Interferon-alfa	11,191	25,808	36999	5.85	abs. dominated	10,236
(branded) Peg-Interferon-alfa with Entecavir (generic) Salvage	27,424	12,348	39772	13.87	abs. dominated	1,579
(branded) Tenofovir	56,019	5,470	61,489	16.90	538,474	3,637
**Cirrhotic HBeAg-negative**						
No Treatment	-	25,293	25,293	4.70		
(generic) Lamivudine	7,122	21,571	28,693	10.79	abs. dominated	2,658
(generic)Lamivudine with Adefovir (generic) Salvage	10,192	15,733	25,925	12.67	79	2,045
(generic) Lamivudine with (branded) Tenofovir Salvage	18,727	10,843	29,570	14.08	2,583	2,099
(generic) Entecavir	20,038	10,848	30,886	14.02	abs. dominated	2,202
(branded) Peg-Interferon-alfa	11,191	26,365	37,556	5.47	abs. dominated	16,036
(branded) Peg-Interferon-alfa with Entecavir (generic) Salvage	25,715	16,122	41,837	11.90	abs. dominated	2,299
(branded) Tenofovir	47,865	10,681	58,546	14.10	1,876,422	4,151

ICER, Incremental Cost-Effectiveness Ratio.

* Calculated as the incremental cost compared to the next-best undominated alternative divided by the incremental QALYs compared to the next-best undominated alternative.

** Calculated as the incremental cost compared to no treatment divided by the incremental QALYs compared to no treatment.

Non-cirrhotic HBeAg-positive patients in the no CHB treatment strategy would likely incur the highest health care costs, and the worst health outcomes, compared to the other strategies. With no antiviral treatment, each HBeAg-positive patient had $12,932 in total healthcare costs, 11.8 QALYs and 65% of them would die of hepatitis-B related liver disease, and 32% will develop HCC over their lifetime. When comparing the different therapies, some therapies appeared better than others. LAM→ADV salvage gave patients 18.0 QALYs at a cost of only $844 per QALY gained. Both ETV and TDF monotherapy offered the highest benefit, 19.0 QALYs, and 7.2 QALYs gained over the no treatment strategy. LAM monotherapy, PEG-IFN monotherapy, and the PEG-IFN→ETV salvage therapy did not appear cost-effective compared to the other therapies, so were all dominated (which means the intervention costs more and is less effective than the comparator). LAM monotherapy provides fewer QALYs at a higher cost-effectiveness ratio than LAM→ADV salvage (known as “dominated by extended dominance” in health-economics). PEG-IFN→ETV salvage therapy had fewer QALYs at a higher cost than ETV alone (known as “absolutely dominated” in health economics). If a low resistance, highly potent drug such as ETV or TDF is used for treatment of HBeAg positive patients who met the treatment criteria, approximately 60% of HBV-related mortality and 31% of HCC can be prevented.

Non-cirrhotic HBeAg-negative patients in the no treatment strategy had $11,735 in costs, 12.2 QALYs and 59% of them would die of hepatitis-B related liver disease, and 30% will develop HCC over their lifetime. When comparing the various therapies, both ETV and TDF offered the highest benefits, 17.7 QALYs and 5.5 QALY gained over no treatment. With no generic TDF and the high cost of brand TDF in the Chinese market, the ICER for generic ETV is $4,066 while the ICER for TDF is $1.5 million per QALY in China. Approximately 49% of HBV-related deaths, and 26% of HCC can be prevented if a low resistance, highly potent drug such as ETV or TDF is used.

If CHB cirrhotic patients are left untreated, 95% would die of HBV related liver disease, and 39% would develop HCC over their lifetime.

For cirrhotic HBeAg-positive cirrhotic patients, LAM→ADV salvage was the lowest cost alternative, it dominated no treatment and LAM therapy alone. LAM→TDF salvage was the next-best option, which added 1.9 QALYs over LAM→ADV salvage therapy at a cost of $15 per QALY. PEG-IFN monotherapy and PEG-IFN→ETV was dominated by ETV monotherapy. Nearly 79% of HBV-related mortality and 33% of HCC in HBeAg-positive patients can be prevented if they are treated with a low resistance, highly potent drug such as ETV or TDF.

For cirrhotic HBeAg-negative cirrhotic patients, LAM→ADV salvage would provide 12.67 QALYs and has the lowest ICER, $79 per QALY. ETV monotherapy or TDF monotherapy had the best health outcomes with 14.0 QALYs, at an additional cost of $2,583, and $1.8 million per QALY, respectively. Approximately 61% of HBV-related mortality and 26% of HCC in HBeAg-negative cirrhotic patients can be prevented if they are treated with a low resistance, highly potent antiviral drug such as ETV or TDF.

### Sensitivity analysis

**Fig 4:**
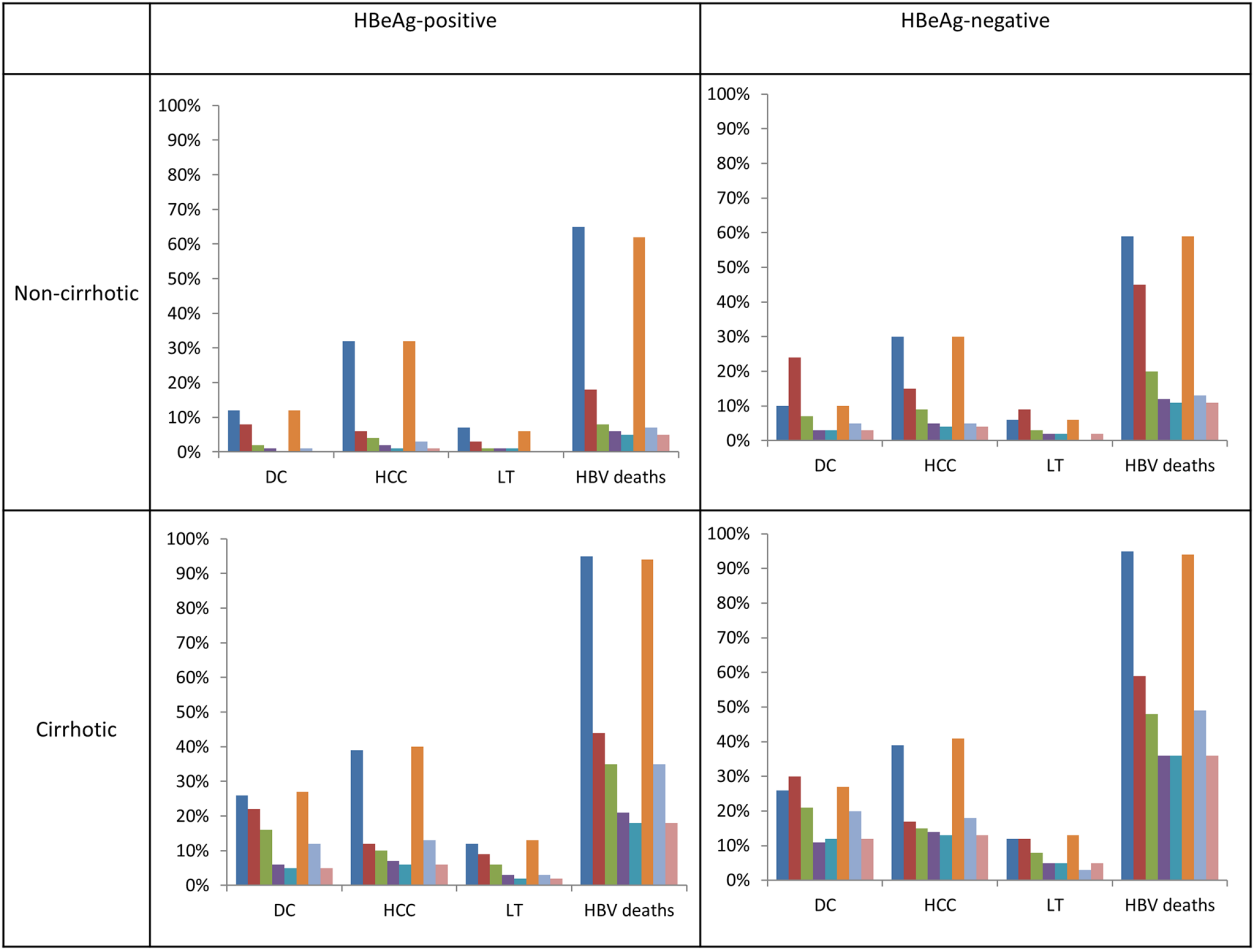
Clinical health outcomes for each strategy stratified by hepatitis B e-antigen (HBeAg) with or without cirrhosis. The bars in order from left to right are: no treatment, LAM, LAM→ADV, LAM→TDF, ETV, PEG-IFN, PEG-IFN→ETV, and TDF.

The results of the probabilistic sensitivity analysis (Fig 4) indicated that unless the cost of TDF drops, among all the therapies, generic ETV is likely the most cost effective therapy for both HBeAg-positive and negative patients with or without cirrhosis in China. For non- cirrhotic HBeAg-positive patients, ETV was 24% likely to be cost-effective at a willingness to pay threshold of $6,800 (1xGDP), and increased to 42% likely to be cost-effective at $20,400 (3xGDP). For the non-cirrhotic HBeAg-negative patients, ETV was 43% likely to be cost-effective at a threshold of $6,800 and further increased to 54% likely to be cost-effective, when compared to other treatments, at a threshold of $20,400. For cirrhotic HBeAg-positive patients, ETV was 32% likely to be cost-effective at a threshold of $6,800 and 55% likely to be cost-effective at a threshold of $20,400. For cirrhotic HBeAg-negative patients, ETV was 45% likely to be cost-effective at a threshold of $6,800 and 48% likely to be cost-effective at a threshold of $20,400. This shows the ETV is most likely to be cost-effective at commonly cited thresholds for cost-effectiveness.

We did an additional analysis on the costs of the two highly potent and low resistance profile drugs, ETV and TDF, to determine the thresholds at which treatment becomes cost-saving (with a willingness to pay (WTP) threshold of $0), highly-cost-effective (WTP $6,800, 1xGDP) and cost-effective (WTP $20,400, 3xGDP) for a national procurement and treatment program in China, compared to no treatment, for all sub-groups (Table 5). We chose ETV and TDF since they were the most effective strategies and had the highest health outcomes in all subgroups. In non-cirrhotic HBeAg-positive patients, ETV and TDF are cost-saving if the drug cost is below $523/year ($44/month), and considered highly cost-effective at $1,008/year ($84/month), and cost-effective at $1,173/year ($98/month). In non-cirrhotic HBeAg-negative patients, ETV and TDF are cost-saving if the drug cost is below $381/year ($32/month), and considered highly cost-effective at $1,313 year ($109/month) and cost-effective at $1,442 ($120/month). For cirrhotic HBeAg-positive patients, ETV and TDF are cost-saving below $899 ($75/month), and considered highly cost-effective at $1,027/year ($85/month), and cost-effective at $1,284/year ($107/month). For cirrhotic HBeAg-negative patients, ETV and TDF are cost-saving below $886 ($74/month), and considered highly cost-effective at $1,120/year ($93/month), and cost-effective at $1,065/year ($89/month).

**Table 5:** Annual Cost Thresholds for Entecavir and Tenofovir.

Population	Cost-saving (WTP $0)		Highly cost-effective (WTP $6,800)		Cost-effective (WTP $20,400)	
	ETV	TDF	ETV	TDF	ETV	TDF
Non-cirrhotic HBeAg-positive	$522	$523	$1,008	$788	$1,173	$986
Non-cirrhotic HBeAg-negative	$378	$381	$1,313	$866	$1,442	$1,051
Cirrhotic HBeAg-positive	$899	$697	$1,027	$861	$1,284	$1,190
Cirrhotic HBeAg-negative	$886	$738	$1,120	$746	$1,065	$762

ETV, entecavir; TDF, tenofovir; WTP, willingness to pay.

Threshold between $6,800–20,400 according to the WHO 1-3x GDP for China.

## Discussion

We examined the cost-effectiveness of treatment for four sub-groups of CHB patients; HBeAg-positive and HBeAg-negative patients with or without cirrhosis. We found neither pegylated interferon with or without salvage therapy were cost effective. Lamivudine monotherapy was also not cost effective. For patients who were previously on lamivudine treatment and developed drug resistance, salvage therapy by switching to generic adefovir was cost saving in cirrhotic, HBeAg-positive patients, and cost effective in cirrhotic, HBeAg-negative patients and in non-cirrhotic patients. Salvage therapy by switching to tenofovir was cost effective in cirrhotic patients and in non-cirrhotic, HBeAg-positive patients.

In our study we found entecavir or tenofovir monotherapy treatment in non-cirrhotic patients would prevent 49–69% of liver related deaths and 26–31% of HCC, and would prevent 61–79% of liver related deaths and 26–33% of HCC in cirrhotic patients. The QALYs gained from entercavir or tenofovir treatment compared to no treatment ranged from 5.5–7.3 for non-cirrhotic patients, and 9.3 to 12.1 for cirrhotic patients. These QALY gains are impressive, since few treatments for chronic non-communicable diseases or chronic infectious diseases result in such a gain in healthy life years. The QALYs gained by treatment is similar to that reported in HIV.

Among the two highly potent, low resistance drugs, entecavir is available as a branded drug at about $196/month (1,204 RMB) or as the less costly generic drug at about $102/month (626 RMB) for CHB treatment. Branded tenofovir is available to the public health system in China for the treatment of HIV/AIDS at the cost of less than $30/month (184 RMB) [53]. Even lower-cost generic tenofovir priced at as little as $5/month (30 RMB) for HIV treatment is available for low-income countries through the Global Fund [53, 54]. Recently, several low-income countries in Asia also have access to generic tenofovir for CHB treatment. Unfortunately, following approval of tenofovir for CHB treatment in China, there is no generic tenofovir available and the price of branded tenofovir for CHB treatment in China is high at $240/month (1,474 RMB), making it out of reach for most infected persons and the public health treatment programs in China.

Our sensitivity analysis indicated that at current prices of the available CHB treatment in China, entecavir is highly likely to be the preferred therapy. According to our threshold analysis, in order for entecavir to be cost-saving, meaning that using this strategy will have the lowest total costs with the best health outcomes, the drug price needs to drop to $671/year ($56/month (344 RMB)) from its current price of $1,229/year ($102/month (626 RMB)) in China. If the price of branded tenofovir for CHB treatment drop to the same level as the price afforded for HIV treatment in China (at less than $30/month), tenofovir treatment would be cost saving.

According to Li et al., a majority of physicians in China still prescribe lamivudine as first line CHB therapy and was prescribed by 54% of physicians in urban areas and 37% of physicians in rural areas, whereas, entecavir was prescribed by 5.4% of physicians in urban and 10% of those in rural areas [55]. Although several studies on the cost effectiveness of CHB treatments in China have been published, our study is the first to include tenofovir, the first to compare nucleoside, nucleotide and interferon therapies and include all four treatment subgroups, and the first to calculate cost thresholds for the most effective treatments. Another strength of our study is that we use age-specific transition estimates taken specifically from recent Chinese cohort studies to capture disease progression in this population as closely as possible. One limitation of our study is that we were unable to find literature on the rate of progression to cirrhosis and HCC for individuals who experience sustained virological response, so we assumed there is a 50% decrease in the rate of disease progression compare with the estimate of patients on treatment without sustained viral response [41, 56].

For a treatment program to be cost-saving, the price of the two highly potent low resistance drugs, entecavir and tenofovir would need to cost between $32-75/month (195–460 RMB). For it to be highly cost-effective or cost-effective, the drug price would need to be lowered to $62-120/month (380–737 RMB). Our study was undertaken to potentially support policy decisions regarding implementation of a national public health treatment program for CHB in China at the population level. This study not only compares the health outcomes but the impact of a reduction in the price of antiviral therapy. Our study could also be adapted to provide similar cost benefit estimates for decision makers in other endemic countries in developing their national viral hepatitis action plan.

## Supporting Information

S1 Table Annual Transition estimates for Pegylated Interferon. (DOCX)

S2 Table Base Case Results (Brand Pricing). (DOCX)
