# Supplementary material for: Cost-Effectiveness and Cost Thresholds of Generic and Brand Drugs in a National Chronic Hepatitis B Treatment Program in China
Source: PLoS One. 2015 Nov 4;10(11):e0139876. doi: 10.1371/journal.pone.0139876 (PMC4633043; doi:10.1371/journal.pone.0139876)
Supplement: S1 Table — (DOCX) [file pone.0139876.s001.docx]

**Supporting Information**

Table S1. Annual Transition estimates for Pegylated Interferon

|  | |  |  |  |
| --- | --- | --- | --- | --- |
|  |  |  |  |  |
| **Initial State** | **Outcome** | **HBeAg+** | **HBeAg-** |  |
| Active chronic hepatitis B | Sustained virological response* | 27 | 15 |  |
|  | 72 weeks post-treatment* | 7 | 2 |  |
|  | Withdrawal* | 2 | 5 |  |
| Cirrhosis | Sustained virological response* | 27 | 15 |  |
|  | 72 weeks post-treatment* | 7 | 2 |  |
|  | Withdrawal* | 2 | 5 |  |
| Sustained Virological Response | Relapse** | 17 | 17 |  |
|  |  |  |  |  |
| * Reference Lau et al., Marcellin et al. and Cooksley et al. [27-29] | |  |  |  |
| ** Estimate from Piratvisuth et al. [30] | |  |  |  |
