# Supplementary material for: Cost-Effectiveness and Cost Thresholds of Generic and Brand Drugs in a National Chronic Hepatitis B Treatment Program in China
Source: PLoS One. 2015 Nov 4;10(11):e0139876. doi: 10.1371/journal.pone.0139876 (PMC4633043; doi:10.1371/journal.pone.0139876)
Supplement: S2 Table — (DOCX) [file pone.0139876.s002.docx]

Table S2. Base Case Results (Brand Pricing)

| **Therapy** | **HBV Antiviral or Interferon Treatment Costs** | **Long-Term Other Healthcare Costs** | **Total Costs ($)** | **Quality Adjusted Life Years** | **ICER compared to next-best therapy ($/QALY)*** | **ICER compared to no treatment ($/QALY)**** |
| --- | --- | --- | --- | --- | --- | --- |
| **Non-cirrhotic HBeAg-positive** | | | | | | |
| No Treatment | 0 | 12,932 | 12,932 | 11.79 |  |  |
| Lamivudine | 14,165 | 5,875 | 20,040 | 16.99 | ext. dominated | 1,367 |
| Lamivudine with Adefovir Salvage | 17,994 | 3,155 | 21,149 | 18.04 | 1,315 | 1,315 |
| Lamivudine with Tenofovir Salvage | 21,964 | 2,267 | 24,231 | 18.73 | 4,419 | 1,628 |
| Entecavir | 51,236 | 1,554 | 52,790 | 19 | 108,359 | 5,528 |
| Peg-Interferon-alfa | 11,191 | 12,174 | 23,365 | 12.35 | abs. dominated | 18,630 |
| Peg-Interferon-alfa with Entecavir Salvage | 54,297 | 2,387 | 56,684 | 18.35 | abs. dominated | 6,670 |
| Tenofovir | 63,720 | 1,518 | 65,238 | 19.02 | 641,651 | 7,235 |
| **Non-cirrhotic HBeAg-negative** | | | | | | |
| No Treatment | 0 | 11,735 | 11,735 | 12.19 |  |  |
| Lamivudine | 12,062 | 14,100 | 26,162 | 13.76 | abs. dominated | 1,757 |
| Lamivudine with Adefovir Salvage | 19,729 | 6,559 | 26,288 | 16.05 | 2,933 | 1,436 |
| Lamivudine with Tenofovir Salvage | 28,531 | 4,071 | 32,602 | 17.5 | abs. dominated | 1,725 |
| Entecavir | 49,844 | 3,709 | 53,553 | 17.71 | 4,066 | 1,681 |
| Peg-Interferon-alfa | 11,191 | 11,613 | 22,804 | 12.34 | ext. dominated | 71,701 |
| Peg-Interferon-alfa with Entecavir Salvage | 54,278 | 4,536 | 58,814 | 17.08 | abs. dominated | 5,426 |
| Tenofovir | 62,032 | 3,640 | 65,672 | 17.73 | 1,492,068 | 3,703 |
| **Cirrhotic HBeAg-positive** | | | | | | |
| No Treatment | 0 | 25,293 | 25,293 | 4.7 |  |  |
| Lamivudine | 10,192 | 16,490 | 26,682 | 13.02 | abs. dominated | 1,920 |
| Lamivudine with Adefovir Salvage | 13,546 | 11,752 | 25,298 | 14.64 | 0 | 1,554 |
| Lamivudine with Tenofovir Salvage | 18,363 | 6,580 | 24,943 | 16.48 | 15 | 1,382 |
| Entecavir | 44,912 | 5,606 | 50,518 | 16.84 | 17,497 | 1,727 |
| Peg-Interferon-alfa | 11,191 | 25,808 | 36,999 | 5.85 | abs. dominated | 10,236 |
| Peg-Interferon-alfa with Entecavir Salvage | 42,217 | 12,348 | 54,565 | 13.87 | abs. dominated | 1,579 |
| Tenofovir | 56,019 | 5,470 | 61,489 | 16.9 | 538,474 | 3,637 |
| **Cirrhotic HBeAg-negative** | | | | | | |
| No Treatment | 0 | 25,293 | 25,293 | 4.7 |  |  |
| Lamivudine | 8,510 | 21,571 | 30,081 | 10.79 | abs. dominated | 2,658 |
| Lamivudine with Adefovir Salvage | 12,178 | 15,733 | 27,911 | 12.67 | 79 | 2,045 |
| Lamivudine with Tenofovir Salvage | 20,606 | 10,843 | 31,449 | 14.08 | 2,583 | 2,099 |
| Entecavir | 38,299 | 10,848 | 49,147 | 14.02 | abs. dominated | 2,202 |
| Peg-Interferon-alfa | 11,191 | 26,365 | 37,556 | 5.47 | abs. dominated | 16,036 |
| Peg-Interferon-alfa with Entecavir Salvage | 38,951 | 16,122 | 55,073 | 11.9 | abs. dominated | 2,299 |
| Tenofovir | 47,865 | 10,681 | 58,546 | 14.1 | 1,876,422 | 4,151 |

ICER = Incremental Cost-Effectiveness Ratio.

* Calculated as the incremental cost compared to the next-best undominated alternative divided by the incremental QALYs compared to the next-best undominated alternative.

** Calculated as the incremental cost compared to no treatment divided by the incremental QALYs compared to no treatment.
